# NMR-Based Tissular and Developmental Metabolomics of Tomato Fruit

**DOI:** 10.3390/metabo9050093

**Published:** 2019-05-09

**Authors:** Martine Lemaire-Chamley, Fabien Mounet, Catherine Deborde, Mickaël Maucourt, Daniel Jacob, Annick Moing

**Affiliations:** 1 UMR1332 Biologie du Fruit et Pathologie, INRA, University Bordeaux, Centre INRA de Nouvelle Aquitaine-Bordeaux, 71 av Edouard Bourlaux, 33140 Villenave d’Ornon, France; mounet@lrsv.ups-tlse.fr (F.M.); catherine.deborde@inra.fr (C.D.); mickael.maucourt@inra.fr (M.M.); daniel.jacob@inra.fr (D.J.); 2 Plateforme Métabolome du Centre de Génomique Fonctionnelle Bordeaux, MetaboHUB, IBVM, Centre INRA de Nouvelle Aquitaine-Bordeaux, 71 av Edouard Bourlaux, 33140 Villenave d’Ornon, France

**Keywords:** fruit development, fruit tissues, seeds, tomato, NMR metabolomic profiling, isoprenoids

## Abstract

Fruit is a complex organ containing seeds and several interconnected tissues with dedicated roles. However, most biochemical or molecular studies about fleshy fruit development concern the entire fruit, the fruit without seeds, or pericarp only. We studied tomato (*Solanum lycopersicum*) fruit at four stages of development (12, 20, 35, and 45 days post-anthesis). We separated the seeds and the other tissues, exocarp, mesocarp, columella with placenta and locular tissue, and analyzed them individually using proton NMR metabolomic profiling for the quantification of major polar metabolites, enzymatic analysis of starch, and LC-DAD analysis of isoprenoids. Pericarp tissue represented about half of the entire fruit mass only. The composition of each fruit tissue changed during fruit development. An ANOVA-PCA highlighted common, and specific metabolite trends between tissues e.g., higher contents of chlorogenate in locular tissue and of starch in columella. Euclidian distances based on compositional data showed proximities within and between tissues. Several metabolic regulations differed between tissues as revealed by the comparison of metabolite networks based on correlations between compounds. This work stressed the role of specific tissues less studied than pericarp but that impact fruit organoleptic quality including its shape and taste, and fruit processing quality.

## 1. Introduction

**Figure 1:**
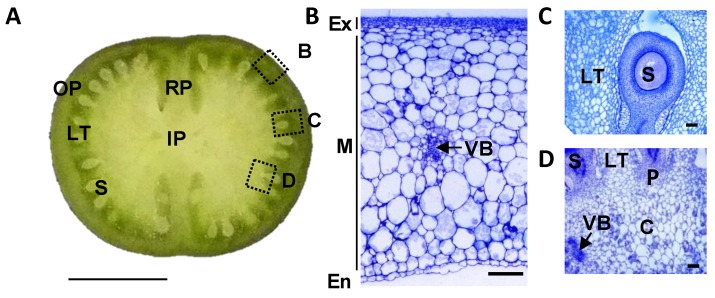
Structure of a tomato developing fruit. (**A**) Equatorial section of Ailsa Craig 12 days post-anthesis (DPA) fruit. The dotted squares indicate the position of fruit zones presented in B–D figures. Bar = 1 cm. (**B**) Outer pericarp of 12 DPA fruit. Bar = 200 µm. (**C**) Locular tissue of 12 DPA fruit. Bar = 200 µm. (**D**) Inner pericarp of 12 DPA fruit. Bar = 200 µm. OP, outer pericarp; RP, radial pericarp; LT, locular tissue; S, seed; IP, inner pericarp; Ex, exocarp; M, mesocarp; En, endocarp; VB, vascular bundles; C, columella; P, placenta.

Fruit is a specialized structure devoted to the protection of the developing seeds in Angiosperms and to their proper dispersal. It results from the differentiation of carpel tissues after ovule fertilization, which leads to the formation of a dry or a fleshy fruit depending on the plant species [1,2]. Accordingly, fruit is a complex organ, composed of several tissues with different implications in seed protection, animal feeding or predator attraction. In tomato (*Solanum lycopersicum* L.), the model plant for fleshy fruits, the fruit is composed of a pericarp which comes from the differentiation of carpel walls and is divided into outer pericarp, radial pericarp or septum, and internal pericarp or columella (Figure 1). The fruit outer pericarp includes three tissues: exocarp or skin, mesocarp, and endocarp. The fruit columella contains the vascular bundles which connect the plant vascular system to the seeds and other fruit tissues, and is delineated at its periphery by the placenta. The fruit locular tissue or gel, which surrounds the seeds at maturity, arises from placental cells a few days after fertilization [3,4]. Some of these tissues are composed of different cell types, like the parenchyma and the vascular cells in columella and in pericarp tissues and are characterized by a heterogeneous differentiation stage and/or size such as in the pericarp tissue [5].

A large diversity in the relative development of the different fruit tissues has been emphasized within the wide range of existing tomato cultivars [6,7] and genetic resources [8]. It is an important trait of individual cultivars. Indeed, processing tomatoes, for which a high solid content is a desirable trait, have generally fruits with highly developed fleshy tissues (pericarp, septum, columella, placenta) and a poorly developed locular tissue [9]. At the opposite, tomato cultivars dedicated to the fresh market are characterized by a wide organoleptic typicity and show a great variability of tissue development, ranging from the cherry tomatoes with a fine pericarp wrapping a large number of seeds bathing in the locular tissue to very fleshy tomatoes with rare locular tissue such as the beefsteak tomato cultivars [6]. In addition to the quantitative considerations on tissue relative proportions within a tomato fruit, the individual metabolic composition of the tissues as well as the physical interactions between tissues are of particular importance with regards to their consequences on fruit global composition and physical properties, such as texture and firmness, and to their relationship with fruit processing and/or fruit taste and nutritional quality. For exocarp tissue, whose interaction with the pericarp cells is related with the fruit peelability trait in processing tomatoes [10], cuticle production may impact fruit organoleptic quality including visual aspect [11].

Most molecular and biochemical studies of fruit development concern the entire fruit, the fruit without seeds, or pericarp only. Studies about the different tissues remain rare. However, each tissue has at least one specific role: exocarp and its cuticle for protection against biotic and abiotic environment [12,13] and seed-dispersing fruit-eaters attraction [14], columella for its vascular bundles and contribution to phloem unloading [15], pericarp for seed-dispersing fruit-eaters rewarding [16], placenta for producing ovule primordia and interfacing maternal tissues and seeds [17], and locular tissue for the prevention of in situ precocious seed germination [18]. The tissue specificity of the exocarp, the locular tissue, and the pericarp was highlighted by molecular studies relying on tissue manual dissection [4,13,19] or laser microdissection techniques [20], giving a limited perspective on fruit tissue transcriptome. Similar approaches have shown that ripening activities are initiated in locular tissue before spreading in other tissues [21,22]. More recently a high-resolution map of tomato fruit transcriptome was established thanks to a combination of manual and laser microdissection coupled with RNA-Seq of all fruit tissues along fruit development and ripening [23], which constitutes a powerful resource to describe the molecular bases of fruit tissue specificity and draw a complete survey of tissular components of fruit transcriptome.

In the same way, whereas a range of biochemical studies in different fruit species have compared flesh and peel composition (e.g., [24,25,26]), only a few compositional studies have characterized all fruit tissues separated using manual dissection. An early work on tomato looked at soluble sugars, vitamin C, and total carotenoids at one stage of development in pericarp, locular tissue, and placenta [27]. A study focused on sucrose and starch showed that starch accumulation was spatially localized in the inner and radial pericarp and columella, and that the outer pericarp and seed locular tissues contained little starch [28]. A work targeted on quercetin, kaempferol, and naringenin flavonoids in exocarp, mesocarp, and seed cavity tissues at three stages of development showed, for instance, that there was significantly more quercetin than kaempferol in exocarp tissue, but that they were present in about equal concentrations in the mesocarp [29]. Besides targeted analyses, mass spectrometry (MS)- or nuclear magnetic resonance (NMR)-based metabolomic approaches have been used more recently to characterize tissular composition in tomato. Specialized metabolite profiling of the vascular attachment region, columella and placenta, epidermis, pericarp, and locular tissue was performed using LC-PDA and LC-MS [30]. This combination of targeted and untargeted analyses revealed that metabolite differences between tissues were more pronounced than differences between ripening stages. Liquid chromatography coupled with diode array detection (LC-DAD) isoprenoid profiling and proton NMR (^1^H-NMR) profiling of polar extracts revealed compositional differences between mesocarp and locular tissue at three stages of fruit growth [19]. This other combination of targeted and untargeted analyses, and also transcriptomics, revealed clear compositional differences between the expanding mesocarp and the locular tissue and specific subsets of genes implicated in key processes of fruit development and metabolism. Although NMR profiling is far less sensitive then MS-based profiling, it allowed absolute quantification of major metabolites.

The combination of biochemical and molecular approaches for fruit pericarp tissue showed that metabolites in the same or related pathways changed in abundance in a coordinated manner during tomato fruit development, revealed a strong relationship between ripening-associated transcripts and specific metabolite groups such as TCA-cycle organic acids and sugar phosphates, and suggested that posttranslational mechanisms dominate metabolic regulation [31]. When other tissues were dissected, such studies showed that the metabolism of each fruit tissue was finely tuned as shown for instance for carotenoid metabolism in peel and flesh of loquat fruit [32], for stilbenes in grape berry skin [33], more generally for specialized metabolism in tomato exocarp [13], and for primary metabolism in tomato pericarp compared to locular tissue [19]. The biosynthesis pathway of ethylene, a hormone crucial for the coordination of fruit development and ripening, has also been shown to be organized and regulated in a tissue specific way [21,22].

The objectives of the present study were to characterize the proportions and compositions of all tissues on the same fruits during fruit development to highlight their similarities and specificities and provide hypotheses about tissue-specific metabolic regulations. This was performed on tomato, a model for fleshy fruits, and using complementary analytical strategies including ^1^H-NMR profiling. This approach allowed showing that the largely studied pericarp tissue represents about half of the entire fruit only, that the composition of each fruit tissue changed during fruit development with common and specific trends, and revealed compositional proximities within and between tissues.

## 2. Results

### 2.1. Pericarp Tissue Represents Only about Half of the Entire Fruit Weight

**Figure 2:**
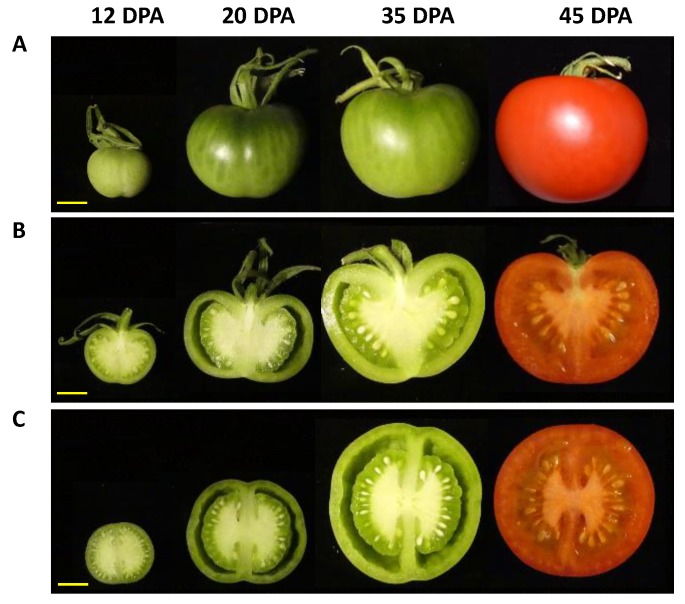
Fruit development of Ailsa Craig tomato from 12 to 45 DPA. (**A**) Whole fruit. (**B**) Fruit longitudinal section. (**C**) Fruit equatorial section. Bars = 1 cm.

**Figure 3:**
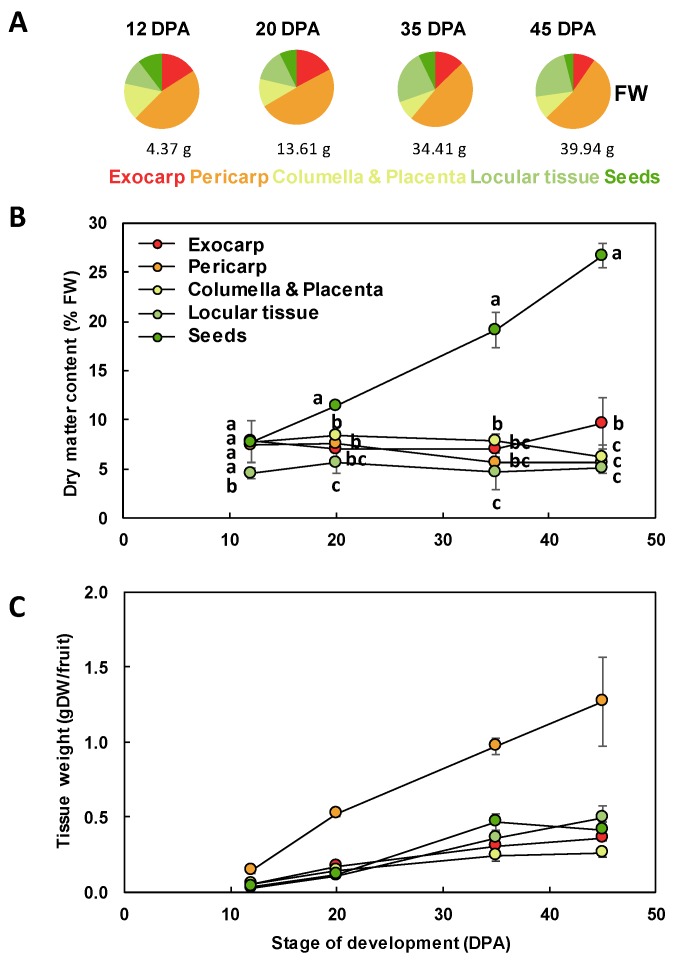
Tissue growth during Ailsa Craig tomato fruit development. (**A**) Tissue proportions along fruit development on a FW basis. The whole fruit mass is indicated below each plot. (**B**) Changes in dry matter content of the different fruit tissues. For each stage, means accompanied by the same letter were not significantly different (Tukey’s studentized test, *P* < 0.05). (**C**) Changes in the dry mass of each fruit tissue. Exocarp in red, Mesocarp with endocarp in orange, Columella with placenta in yellow, Locular tissue in light green, Seeds in dark green. Mean ± SD of 3 replicates per stage. Vertical bars represent SD.

The different tissues of a tomato fruit, exocarp, mesocarp, endocarp, columella and placenta, and the locular tissue surrounding the young seeds, are visualized in Figure 1 at 12 DPA. Ailsa Craig is a middle-sized tomato adapted for greenhouse as well as field culture. In our culture conditions in a greenhouse, mature fruits reached approximately 40 g with a 45 mm diameter and fruits presented a round shape, sometimes slightly flattened (Figure 2). The fruit weight increased from 4.4 to 39.9 g fresh weight (FW) between 12 and 45 DPA (Figure 3). The pericarp was thin, but its thickness increased from 1.5 mm to 5.3 mm between 12 and 45 DPA (data not shown). For all stages of development, pericarp represented about half of the fruit fresh weight (Figure 2A,B; Figure 3A). Ailsa Craig fruits were also characterized by the presence of a large columella at 12 DPA, which filled almost all the fruit inside (Figure 2B,C). However, the proportion of this tissue decreased along fruit development from 16.0% at 12 DPA to 10.0% at 45 DPA (Figure 2B,C; Figure 3A). Similarly, the proportion of the exocarp tissue decreased regularly from 15.9% at 12 DPA to 9.6% at 45 DPA (Figure 3A). On the opposite, the proportion of the locular tissue increased along fruit development (11.3% to 23.3%) in agreement with the fact that it is a newly differentiated tissue after fruit fertilization (Figure 3A). As a consequence, at 45 DPA, the locular tissue was the second more developed tissue within Ailsa Craig fruit.

In addition to changes in the relative proportion of the different fruit tissues, this work also revealed that the dry matter content within the fruit was dependent on the tissue considered and on its developmental stage (Figure 3B). As expected, from 20 to 45 DPA, the seeds presented a significantly higher dry matter content than the other tissues. At 12 DPA, the dry matter content of the locular tissue was significantly lower than that of all the other tissues. At 45 DPA, the exocarp dry matter content was significantly higher than that of columella, locular tissue, or exocarp. Because of these differences, for comparison between tissues, we thereafter expressed our data on a dry weight (DW) basis for growth and biochemical composition. The growth of the different tissues expressed on a DW basis showed similar patterns with a regular increase except for seeds that plateaued at 35 DPA (Figure 3C).

### 2.2. The Composition of Each Fruit Tissue Changes During Fruit Development with Common and Specific Trends

**Figure 4:**
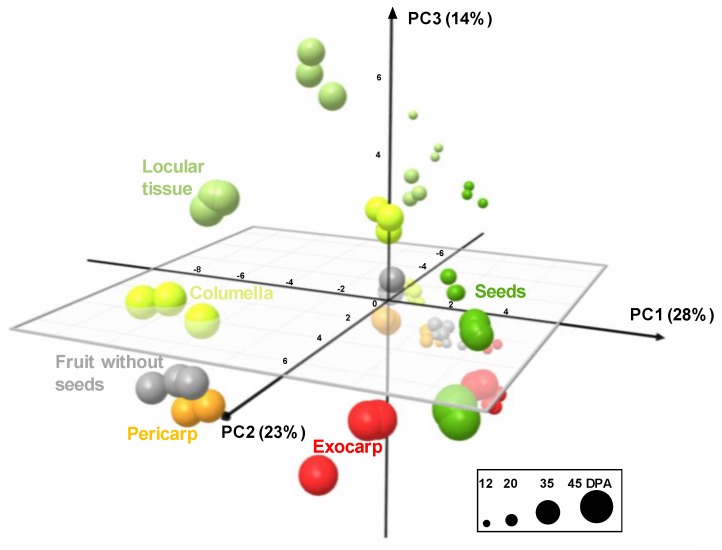
PCA of absolute values of 39 metabolites and starch measured by proton nuclear magnetic resonance (^1^H-NMR), liquid chromatography coupled with diode array detection (LC-DAD), or enzymatic analyses in five tomato fruit tissues and fruit without seeds at four stages of development, and expressed on a DW basis. 3D scores plot for PC1, PC2, and PC3. Exocarp in red, mesocarp with endocarp in orange, columella with placenta in yellow, locular tissue in light green, seeds in dark green, and fruit without seeds in light grey.

The analytical strategies chosen allowed the absolute quantification of 39 metabolites, including six soluble sugars, four organic acids, 14 amino acids or other amino compounds, 11 isoprenoids, and starch in all tissues at four stages of development (Figure S1). In order to get an overview of the compositional differences, a principal component analysis (PCA) was performed on all tissues and also in fruit without seeds. The first three components explained about 65% of total variability. The PCA scores plot (Figure 4) showed that each tissue followed its own compositional trajectory during fruit development. Fruit without seed samples were intermediate between pericarp and columella samples. The first principal component (PC1, about 28% of total variability) separated the exocarp and the seeds from the other tissues. PC2 differentiated the stages of development with parallel trends for all tissues. PC3 tended to separate locular tissue from the other tissues. Overall, several global compositional differences between tissues for a given stage seemed to be of the same order of magnitude as those between stages for a given tissue.

**Figure 5:**
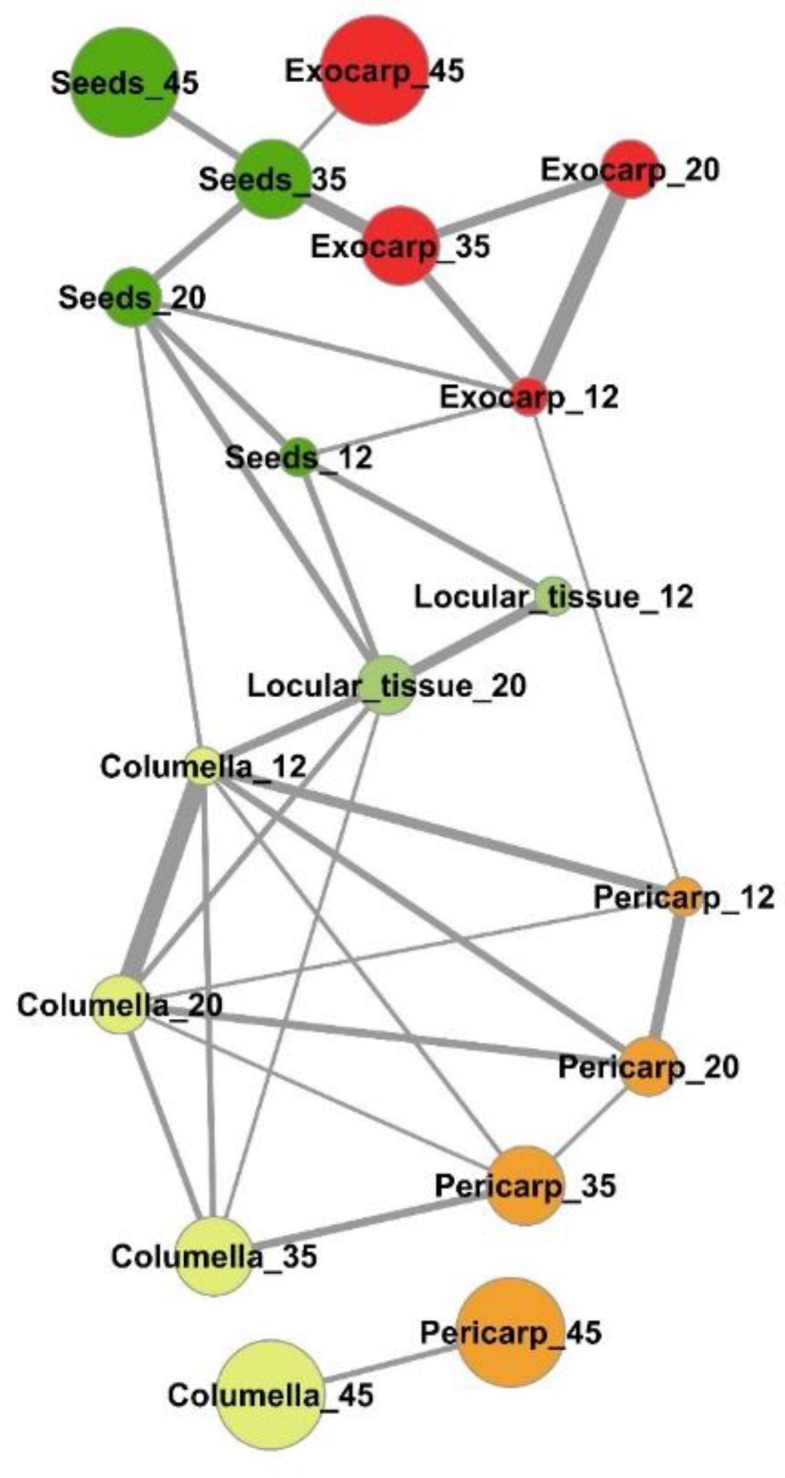
Mapping of compositional distances between sample means per tomato fruit tissue and stage of development based on the absolute contents in 39 metabolites and starch. Euclidian distances (edges) calculated using mean centered and unit-variance scaled data of five tissues at four stages of development (nodes). Only sample mean pairs with distances lower than 0.5 are mapped. All distances are indicated in Figure S2. Exocarp in red, Mesocarp with endocarp in orange, Columella with placenta in yellow, Locular tissue in light green, Seeds in dark green. The node size is proportional to the fruit stage of development. The edge width is inversely proportional to the distance value.

An estimation of the compositional distance between the sample means (Euclidian distance based on all compounds, Figure S2) confirmed the proximity of consecutive developmental stages of a given tissue for exocarp, pericarp, columella, and seeds. The mapping of the shortest compositional distances between sample (Figure 5, Euclidian distance <0.5) revealed a general compositional proximity between successive stages of the same samples during early fruit development (12–35 DPA stages). In addition, it revealed a compositional proximity between columella and pericarp tissues at 12, 20, 35, and 45 DPA, and a short compositional distance between the locular tissue at 20 DPA and columella at 12, 20, and 35 DPA. Seeds at 12 DPA and 20 DPA presented also compositional similarities at the same stages with the locular tissue, and more surprisingly similarities with exocarp tissue at 12 and 35 DPA.

**Figure 6:**
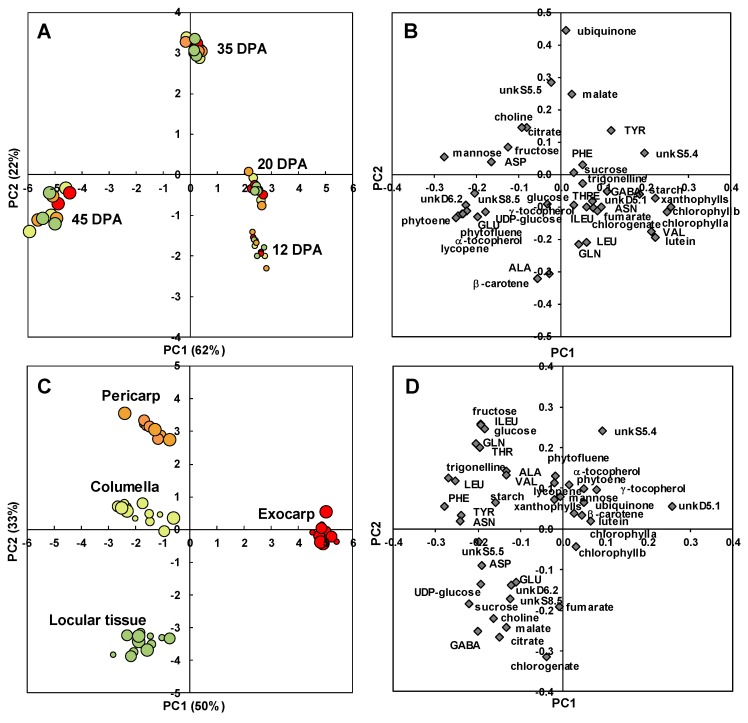
ANOVA-PCA of absolute values of 39 metabolites and starch measured by ^1^H-NMR, LC-DAD or enzymatic analyses in four tomato fruit tissues at four stages of development. (**A**) PC1 × PC2 scores plot for developmental stage effect. (**B**) PC1 × PC2 loadings plot for developmental stage effect. (**C**) PC1 × PC2 scores plot for tissue effect. (**D**) PC1 × PC2 loadings plot for tissue effect. Exocarp in red, Mesocarp with endocarp in orange, Columella with placenta in yellow, Locular tissue in light green.

A PCA after variance analysis (ANOVA-PCA) approach was then chosen to highlight common and specific metabolite trajectories between four tissues: the exocarp, pericarp, columella, and locular tissues. The scores plot for stage effect (Figure 6A) clearly separated the four stages of development with the first two stages on the positive side, and the last stage on the negative side of PC1. A comparison of the scores and loadings plots (Figure 6B) revealed the compositional differences between stages. Samples at 12 and 20 DPA were characterized by higher contents in chlorophylls as expected, but also xanthophylls, lutein, and valine. Samples at 35 DPA were characterized by higher contents in malate, ubiquinone, and unknown compound unkS5.5. Samples at 45 DPA were characterized by higher contents in mannose, five isoprenoids (phytoene, lycopene, α- and γ-tocopherol, phytofluene), glutamate, unkD6.2 and unkS8.5 unknown compounds. UnkD6.2 compound was identified using dedicated 1D and 2D NMR experiments (Text S1) as adenosine 5’-monophosphate (AMP). As our extraction protocol was not dedicated to preserve adenosine-phosphate compounds, unkD6.2 analyte may correspond to the sum of mono-, di-, and tri- adenosine-phosphates.

**Figure 7:**
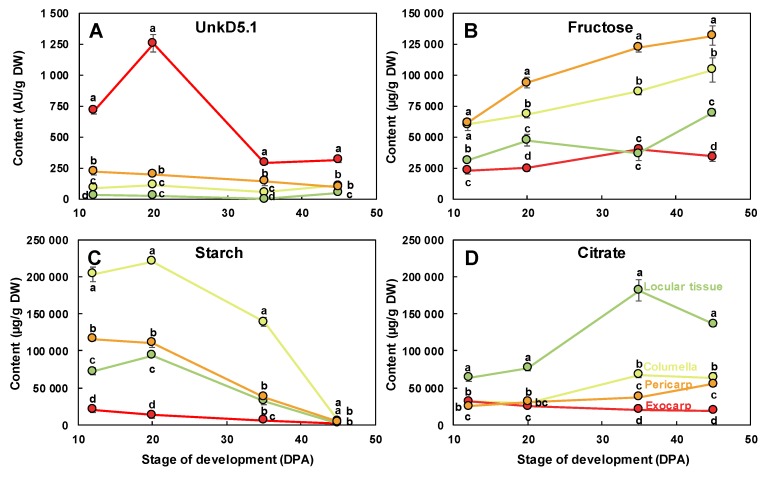
Changes of representative compounds in each of the four studied fruit tissues. (**A**) UnkD5.1 and (**B**) fructose measured using ^1^H-NMR, (**C**) starch measured using enzymatic analysis, and (**D**) citrate measured using ^1^H-NMR in the dissected tissues during tomato fruit development and expressed on a dry weight (DW) basis. Exocarp in red, mesocarp with endocarp in orange, columella with placenta in yellow, and locular tissue in light green. Mean ± SD of 3 replicates per stage. Vertical bars represent SD. For each stage, means accompanied by the same letter were not significantly different (Tukey’s studentized test, *P* < 0.05). AU, arbitrary units.

The scores plot for tissue effect (Figure 6C) clearly separated the four tissue effects with the exocarp on the positive side and the other tissues on the negative side of PC1. PC2 separated the pericarp from columella and locular tissue effects. A comparison of the scores and loadings plots (Figure 6D) revealed the compositional differences between tissues independently of stages. The exocarp samples were characterized by higher contents in an unknown compound presenting a doublet at 5.1 ppm with a small coupling constant of 0.9 Hz. This compound was tentatively identified using dedicated 1D and 2D NMR experiments (Text S1). Although the compound could not be unambiguously identified, the 1D and 2D spectral information suggest that it is a compound with a moiety of α-rhamnosyl. The pericarp samples were characterized by higher contents in fructose, glucose, isoleucine, threonine and glutamine. The columella samples were characterized by higher contents in starch. The locular tissue samples were characterized by higher contents in chlorogenate, citrate, malate, γ-aminobutyrate (GABA) and choline. The changes of a representative compound significantly enriched in each of the four studied tissues are presented in Figure 7: unkD5.1 for the exocarp (Figure 7A), fructose for the pericarp (Figure 7B), starch for the columella (Figure 7C) and citrate for the locular tissue (Figure 7D).

### 2.3. Several Metabolic Regulations Differ Between Tissues as Revealed by Metabolite Network Comparisons

**Figure 8:**
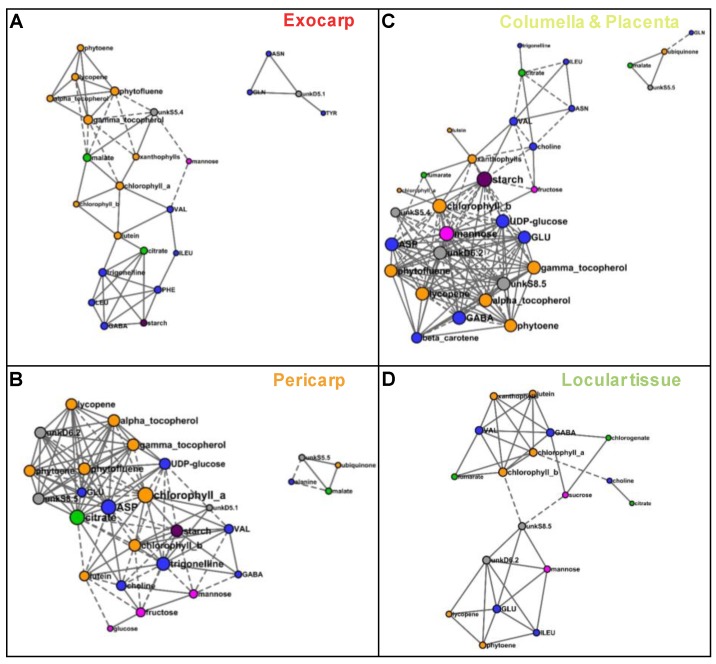
Correlation networks based on the absolute values of 39 metabolites and starch measured by ^1^H-NMR, LC-DAD, or enzymatic analyses and expressed on a DW basis, for each of four tissues. Correlations were filtered based on their significance with a false discovery rate correction (q < 0.001) and visualized using Cytoscape for the sub-networks comprising more than three nodes. (**A**) Exocarp. (**B**) Mesocarp with endocarp. (**C**) Columella with placenta. (**D**) Locular tissue. Metabolites are colored according to their biochemical family: sugars in pink, organic acids in green, amino acids and other amino compounds in blue, isoprenoids in orange, and starch in purple. Full line, positive correlation; dashed line, negative correlation. The size of each node is proportional to its number of edges.

In order to compare metabolite co-regulations between tissues, we reconstructed correlation networks between compounds based on Pearson coefficients (*P* < 0.001 threshold with FDR correction) for each of four tissues. The network numbers of nodes (compounds) and edges (connections between compounds), average number of neighbors, densities, and hubs are presented in Table 1. The larger network was that of columella with 35 compounds. For each tissue, the number of positive connections was higher than that of negative ones. The average number of neighbors and network densities for exocarp and locular tissue were lower than that of pericarp and columella. The sub networks comprising at least four nodes are detailed in Figure 8 for each tissue. For the two exocarp sub-networks comprising 24 nodes (Figure 8A), the node with a number of connections superior or equal to 8 (considered as a hub) was γ-tocopherol. For the two pericarp larger sub-networks comprising 27 nodes (Figure 8B), the three nodes with the highest number of connections were chlorophyll a, aspartate, and citrate (16, 15, and 15 connections, respectively). For the columella larger two sub-networks comprising 31 nodes (Figure 8C), the three nodes with the highest number of connections were starch, mannose and chlorophyll b (18, 16, and 17 connections, respectively. For the locular tissue larger sub-network comprising 18 nodes (Figure 8D), the node with the highest number of connections was chlorophyll b with seven connections only.

Based on the significant correlations observed between metabolites, the metabolite co-regulations therefore appear to depend on the fruit tissue considered. Tighter co-regulations between all compound families seemed to characterize the pericarp and columella compared to the exocarp and locular tissues. The metabolite nodes with the higher number of connections also depended on the tissue. Although chlorophyll a and chlorophyll b were directly correlated in the four tissues, chlorophyll a was a hub in pericarp network, whereas chlorophyll b was one in columella network. Starch had more than 10 connections in pericarp and columella only.

## 3. Discussion

Metabolism analysis at a fruit-wide level may veil tissue-specific phenomena. Here, hand-dissection of four fruit tissues, the exocarp, mesocarp with endocarp, columella with placenta, locular tissue, and seeds, allowed measuring their relative importance on a weight basis, estimating their compositional trajectories and similarities along fruit development. Our compositional data acquired in all tissues along fruit development and ripening are in global agreement with data obtained on a more limited range of fruit tissues or stages [28,30,34,35]. Their mining allowed providing hypotheses about the relations between the regulation of tissue metabolism and the specific role of each tissue for fruit growth and seed dispersal.

### 3.1. Changes in Tissue Proportions Impact Fruit Organoleptic Quality

Characterization of fruit tissue proportion and composition in a particular tomato cultivar is a first step towards understanding the input of the different fruit tissues to fruit final organoleptic quality. In the present work, characterization of Ailsa Craig fruit tissues obviously showed that despite their spatial proximity these tissues presented particular compositional differences, and consequently may contribute differently to tomato organoleptic quality. Indeed, the pericarp tissue was enriched in fructose, glucose, isoleucine, threonine, and glutamine, whereas the locular tissue was enriched in chlorogenate, citrate, malate, GABA and choline, and the columella was enriched in starch. Citrate was the major metabolite in the locular tissue at 45 DPA contributing to its vacuolar osmolarity in relation with its large cell size [19]. These compositional differences modify the sugar over acid ratio which has a direct impact on fruit taste [36]. Starch as a transitory carbohydrate reserve may contribute to mature fruit sugar contents [37]. Changes in amino acids such as isoleucine may also indirectly have an impact on tissue taste as it is a precursor of volatile aroma compounds [38]. Increases in the content of GABA with potential signaling roles for growth and development through membrane signaling and the molecular mechanisms of which have been reviewed recently, may be interesting for human health also [39], and possibly achieved through an increase of locular tissue abundance.

Some metabolite families relevant for fruit organoleptic quality were missing in the present study, including specialized metabolites and cell-wall polymers, and lipids as previously analyzed in flesh and seed only for fatty acid methyl esters [40]. A detailed analysis of lipids in apolar extracts would be of interest as several lipids are aroma precursors [38]. Tissue differences for aromas have been rarely studied, but volatile acetates have been analyzed specifically in peel or flesh of apple [41]. Concerning specialized metabolites, the glycoalkaloids α-tomatine and dehydrotomatine were shown to have about ten times higher contents in the locular gel than in the skin and be present as traces in the pulp in one tomato cultivar [35], and capsaicinoids were shown to be synthesized in the placental epidermis of pungent pepper [42]. Cell-wall polymer composition plays a major role in fruit texture together with fruit and tissue anatomy and cell turgescence as well as osmotic pressure [43]. Here, we analyzed the water content of the different fruit tissues, and showed slight variation of the dry matter content between fruit tissues at 12 DPA and 45 DPA. Data describing the turgor or osmotic potential of different fruit tissues as done for cherry skin and pulp [44], and their cell-wall compositions as done for flesh, parenchyma cells, stone cells, and skin of ripe pear fruit [45], remain rare. In tomato fruit, cell-wall composition has been mainly characterized for the pericarp tissue [31,46]. However, transcriptomic data, fruit macroscopic observations as well as the differential staining of cell-walls in the different tissues strongly suggest discrepancies of the fruit tissues in term of their physical properties [4,23,47]. In particular, the locular tissue seems to have thin cell-walls compared to other fruit tissues in the present work. In contrast, tomato fruit exocarp tissue has small cells with cutinized cell wall in the epidermal cell layers [48], which gives elasticity properties to the fruit surface.

The tissue proportions may also be linked to fruit shape and size. However, their changes over time are rarely measured in fleshy fruits. The wide diversity in fruit size and shape observed in tomato cultivars has been largely studied because of the participation of these traits in fruit yield. This allowed identifying alleles conferring increased fruit size like *fw2.2* [49], carpel number *fasciated* and *locule number* [50,51]. Similarly, the genetic origin of the elongated tomatoes cultivars, has been attributed so far to *SUN, OVATE,* and *fs8.1* genes [6,52,53] and the divergence of processing tomatoes, characterized by their high soluble solid content and increased firmness, was shown to originate from the selection of the entire tomato chromosome 5 [8]. The wide diversity of tomato cultivars is also a collection of tissue proportion variants: fruit with a high locule number have generally a hypertrophied columella corresponding to the fusion of the numerous locules, and elongated tomatoes generally present a poorly developed locular tissue and only a few seeds [6,54]. Tomato breeding has selected the latter type of tomato cultivars for the processing market. However, so far, the global effect of changes in fruit tissue proportions on fruit global organoleptic quality has not been studied per se, except when looking for fruit firmness trait which integrates all fruit tissue individual properties, their proportion and relative organization inside the fruit [55].

### 3.2. Several Spatially-Close Tissues Seem to Have Closer Metabolic Patterns

Despite the tissue specific metabolic characteristics highlighted above, an ANOVA-PCA also revealed that all tissues presented parallel patterns during fruit development for several metabolites, for instance higher contents in chlorophylls, two isoprenoids and valine at the first two stages, or higher contents in several other isoprenoids, glutamate and mannose at 45 DPA, suggesting generic metabolic or regulatory processes for these particular metabolites. However, based on compositional Euclidian distances and reconstructed correlation networks, spatially-close tissues seemed to have closer metabolic signatures or metabolite patterns.

In this way, the columella and pericarp tissues presented similar compositional trends at all stages of development. This outcome is in agreement with the close transcriptomic profiles of these tissues [23], and could result from the common ontogeny of both tissues, columella resulting from the fusion of the carpel walls together with a column extending from the central part of the floral meristem [17,56]. In the same way, the locular tissue and columella with placenta tissues presented similar compositional trends at 20 DPA, relevant with the available transcriptomic data [23], and with their ontogeny, locular tissue resulting from the differentiation of the placenta cells after fertilization [3,4]. At the younger stages only, when their water contents were the closest, seed and locular tissue presented similar compositional trends. This result is consistent with the transcriptomic data which showed closest expression profiles for both samples at early stages than later on during fruit development [23]. At the moment, this result is difficult to handle considering the different origins and structural features of locular tissue and seeds. On one hand, it could be related to common cell differentiation processes within locular tissue and seed at these early stages [3,57], and/or one can suggest that the role of locular tissue in maintaining the developing seed in a favorable environment relies on close metabolic composition of both tissues [18]. On the other hand, a possible partial cross-contamination between locular tissue and seed cannot be excluded, as precise manual dissection of these two tissues was difficult at that stage. A last similar compositional trend was found between seeds and the exocarp tissue at 12, 35, and 45 DPA, which is difficult to explain at the moment, due to the lack of usable transcriptome data permitting to compare both sample types, and due to the wide ontogenic differences between the exocarp and the seed. However, both samples shared common features such as the presence of small cells and specialized metabolisms namely dealing with lipid metabolism for cuticle or reserves, the metabolism of which is connected with central metabolism [58].

In addition to these similar compositional trends, the reconstructed correlation networks presented hubs that were common for two tissues. Starch was a hub in both the pericarp and columella networks, in agreement with a previous biochemical study about sucrose to starch metabolism during tomato development [28]. Chlorophylls and tocopherols were major hubs in several tissue, which suggests the importance of plastid metabolism in all tissues possibly in relation with stress tolerance processes [59,60]. Chlorophyll a was a major hub in pericarp, whereas chlorophyll b was one in columella. Although both chlorophylls a and b are part of light harvesting complexes that contribute to photosynthesis in pericarp, they also play other roles in fruit tissues in relation with ripening [61]. Chlorophyll b may play a particular role in the columella tissue perceiving less light.

Based on the reconstructed correlation networks, metabolite co-regulation seemed higher in pericarp and columella than in the other two tissues. The columella tissue harbors the vascular bundles that connect the fruit sink to the source leaves and the maternal part of the fruit to the seeds. It therefore plays a crucial role for phloem unloading, transient storage of carbon in the form of starch, and (re)distribution of imported carbon to the other fruit tissues. Structural starch characteristics were similar in tomato fruit pericarp and columella except for granule size and crystallinity that were slightly higher in columella [62]. A pioneering work about molecular tissular differences showed that sucrose synthase mRNA was differentially localized in tomato fruit, being most abundant in the mesocarp cells adjacent to the placenta, the columella, and the cells surrounding the vascular bundles [63], suggesting intense sugar metabolism in these cells. The lowest metabolite co-regulation observed in exocarp and locular tissues may be linked with the absence of direct connection with vascular tissues that could limit their nutrient supply, and also a higher specialization of these two tissues.

### 3.3. More Spatial Transcriptomic and Metabolomic Studies of Fruit are Needed

In the present work, we dissected the different tissues and then analyzed them individually. For a more detailed characterization of the compositional differences between tissues, spatial metabolomics and transcriptomics would help. Tissue-specific transcriptomic analyses in fruit have been performed in several species and with different objectives. A transcriptomic analysis of cucumber exocarp [12] showed that the transcripts predominantly expressed in the peel were consistent with fruit surface associated functions including photosynthesis, cuticle production, response to the environment, and defense. A similar approach in tomato with laser microdissection focusing on five tissues of the pericarp [20] provided insights about metabolic and regulatory specialization in this tissue and particularly cuticle formation. The combination of laser dissection and high-throughput RNA sequencing improved spatial resolution and revealed tissue-specific regulatory programs during early tomato fruit development [64]. Later, Shinozaki et al. [23] published a detailed comprehensive tomato fruit transcriptome atlas of several cell/tissue types, including pericarp and five internal tissues. They showed that about half of the genes were ubiquitously expressed in the six fruit tissues, and revealed considerable spatial variation in the expression of metabolic genes involved in the accumulation of compounds that are nutritionally important such as GABA. The entire potential of the corresponding data has not been fully exploited yet. Moreover, more combination of transcriptomic data with other omic data, such as proteomic and metabolomic are needed.

Spatial metabolomic approaches in fruit have been less used than spatial transcriptomic ones so far. Biopsy sampling of locular gel and pericarp tissue [34] has been used to repeatedly quantify major sugar and organic acid levels over time and parameterize a metabolic model for a post-harvest study. Chemical shift imaging, an NMR method with spectra of voxels in the outer pericarp and columella, was used to investigate spatial-temporal changes in sugar and lycopene contents of tomato fruit during ripening [65]. Such approaches may for instance contribute to detail the specific metabolic roles of placenta compared to columella tissues that were not separated in the present work. MS imaging would also be of interest for the characterization of tomato fruit tissues. Indeed, matrix-assisted laser desorption/ionization mass spectrometric imaging (MALDI-MSI) of *Capsicum* fruits was used to study the localization of capsaicin alkaloid and has shown that its content was higher in placenta than in pericarp tissue [66], and to shed light on the spatial distribution of flavonoids during strawberry fruit development [67]. In the same way, desorption electrospray ionization-mass spectrometry (DESI-MS) imaging revealed the spatial distribution of chlorogenic acids and sucrose across coffee bean endosperm [68].

## 4. Materials and Methods

### 4.1. Plant Material

Fifteen tomato (*Solanum lycopersicum* cv. ‘Ailsa Craig’) plants were grown in a growth chamber and fruits were harvested and samples prepared as described in Mounet et al. (2009) at four stages of development. The last stage at 45 DPA corresponded to red-ripe stage. Three pools (biological replicates) of six (20, 35, and 45 DPA) or 12 (12 DPA) fruits were harvested. One-quarter of each fruit was used to constitute the “fruit without seed” sample. The rest of each fruit was separated into four tissue types: exocarp, mesocarp with endocarp (named “pericarp” in the present work), columella with placenta (named “columella” in the present work), locular tissue, and seeds and rapidly weighed to determine the proportions of the different tissues and frozen in liquid nitrogen. For biochemical analyses, the tissue issued from 6 or 12 fruits were pooled, ground in liquid nitrogen, and stored at −80°C until use. Frozen samples were lyophilized before untargeted or targeted metabolite analyses. Dry matter content was determined by recording the weight of the grounded tissue before and after lyophilization.

### 4.2. Cytological Study

Fruits were collected at 12 DPA stage, cut (approximately 0.3 to 0.6 mm-thick fruit pieces), and rapidly fixed for 2 h in ethanol-acetic acid (3:1, v/v) at room temperature. The samples were rinsed three times in 70% ethanol, dehydrated by an ethanol series, and embedded in Technovit 7100 (Kulzer GmbH, Hanau, Germany). Sections (3 µm) obtained with glass knives were stained with 0.04% (w/v) toluidine blue and photographed using a Axio Zoom V16 Microscope (Zeiss, Oberkochen, Germany) coupled to a AxioCam 105 RGB camera (Zeiss, Oberkochen, Germany).

### 4.3. Metabolite Analysis

^1^H-NMR profiling of polar extracts for the quantification of major polar metabolites (including soluble sugars, organic and amino acids, and quaternary amines), enzymatic analysis of starch in pellets, and LC-DAD analysis of isoprenoids in methanol/Tris buffer/chloroform extracts were carried out as described in Mounet et al. [19,40]. For ^1^H-NMR profiling, precautions have been taken to allow absolute quantification of metabolites using the addition of ethylene diamine tetraacetic acid sodium salt solution to improve the spectra resolution especially in the citrate region, adequate choice of the NMR acquisition parameters, and an electronic reference and calibration curves for quantification [19,40]. The concentration of each organic or amino acid was expressed as g of the acid form per g DW and the concentration of unknown compounds was calculated hypothesizing that resonance corresponded to one proton and using an arbitrary molecular weight of 100 Da. For isoprenoids, “xanthophylls” refers to the sum of zeaxanthin and violaxanthin. Compositional data of 12, 20 and 35 DPA in pericarp and locular tissues have already been published in Mounet et al. [19] but several isoprenoids are added here. Compositional data of seed and fruit without seed at all stages have already been published in Mounet et al. (2007). The compositional data of exocarp and columella with placenta at all stages, and of all tissues at 45 DPA have not been published previously. For ^1^H-NMR, unknown compounds were named using the mid value of the chemical shift and the multiplicity of the corresponding resonance group (i.e., unknownD5.1 for a doublet at 5.10 ppm). Additional 1D and 2D NMR experiments (COSY, HSQC, HMBC) were performed on two supplementary pericarp samples (Supplementary Material Text S1) for the tentative annotation of unknown compounds.

### 4.4. Data Analysis

For growth parameters and individual metabolites, mean ± standard deviation (SD) were calculated from three replicates. For all biochemical analyses, two extractions (technological replicates) were prepared per biological replicate, then the mean of three biological replicates was calculated. For univariate analyses, variance analysis (ANOVA) was used for compositional data and a mean comparison test was used for tissue dry matter content. For multivariate analyses, principal component analysis (PCA) and ANOVA-PCA [69] were performed using BioStatFlow web tools based on R scripts (biostatflow.org), on data mean-centered and scaled to unit variance. The PCA 3D plot was done with XLSTAT (v2012.3.01, Addinsoft, Paris, France). For ANOVA-PCA, covariations were separated into stage and tissue effects and their interaction using ANOVA. The covariances for each effect were combined with the error and subjected to PCA. Euclidian distances between means per tissue and stage were calculated using Multi experiment viewer v4.9 [70] on compositional data mean centered and scaled to unit variance and mapped using Cytoscape software version 3.5 ([71]; http://www.cytoscape.org/). Correlation networks were based on pairwise Pearson correlation coefficients. Network cartography was done with variable filtering based on false discovery rate correction (q < 0.001), and Fruchterman layout, using R scripts in BioStatFlow and Cytoscape software version 3.5.

## 5. Conclusions

Absolute quantification of compounds through ^1^H-NMR profiling and LC-DAD analyses allowed combining already published data and new data acquired with the same protocols, and in addition may allow further comparisons. The separation and quantitative metabolomic characterization of each of the different tissues of tomato fruit revealed compositional similarities with parallel trends along development, but also content differences that may affect fruit organoleptic quality including the sugar over acid ratio. Several spatially-close tissues seemed to have closer metabolic patterns possibly linked with their ontogeny. As pericarp may represent about half of the fruit tissues only, more combined spatial transcriptomic and metabolomic studies of fruit are needed to describe tissue-specific metabolic regulations and better decipher how the development of the entire fruit integrates such tissular specificities.

## Figures and Tables

**Table 1:** Parameters of the correlation networks for four tomato fruit tissues based on the absolute contents of 39 metabolites and starch along fruit development measured by ^1^H-NMR, LC-DAD, or enzymatic analyses and expressed on a DW basis. For each tissue, correlations were filtered based on their significance with a false discovery rate correction (q < 0.001). The larger sub-networks comprising more than three nodes are presented in Figure 8.

Tissue	Number of Nodes	Number of Edges (Negative/Positive)	Average Number of Neighbors	Network Density
Exocarp	24	51 (10/41)	4.3	0.185
Pericarp	29	114 (37/77)	7.9	0.281
Columella	35	144 (54/90)	8.2	0.242
Locular tissue	26	46 (3/43)	3.5	0.142

## Supplementary Materials

The following are available online at https://www.mdpi.com/2218-1989/9/5/93/s1, Text S1: Tentative annotation of unknown metabolites unkD5.1 and unkD6.2, Figure S1: Changes of 40 compounds measured by ^1^H-NMR, LC-DAD or enzymatic analyses in the different tissues during tomato fruit development and expressed on a DW basis, Figure S2: Compositional distances between sample means per tomato fruit tissue and stage of development based on the absolute contents in 39 metabolites and starch.
