# Supplementary material for: NMR-Based Tissular and Developmental Metabolomics of Tomato Fruit
Source: Metabolites. 2019 May 9;9(5):93. doi: 10.3390/metabo9050093 (PMC6571556; doi:10.3390/metabo9050093)

Supplementary Figure S1: Changes of 40 compounds measured by ^{1}H-NMR, LC-DAD or enzymatic analyses in the different tissues during tomato fruit development and expressed on a DW basis. Fruit tissues in circle symbols with Exocarp in red, Mesocarp with endocarp in orange, Columella with placenta in yellow, Locular tissue in light green, Fruit without seeds in grey with dashed line. Seeds with triangle symbols and in dark green. Mean ± SD of 3 replicates per stage. Vertical bars represent SD.

Article title: NMR-based tissular and developmental metabolomics of tomato fruit. *Authors*: Lemaire-Chamley M., Mounet F., Deborde C., Maucourt M., Jacob D., Moing A.

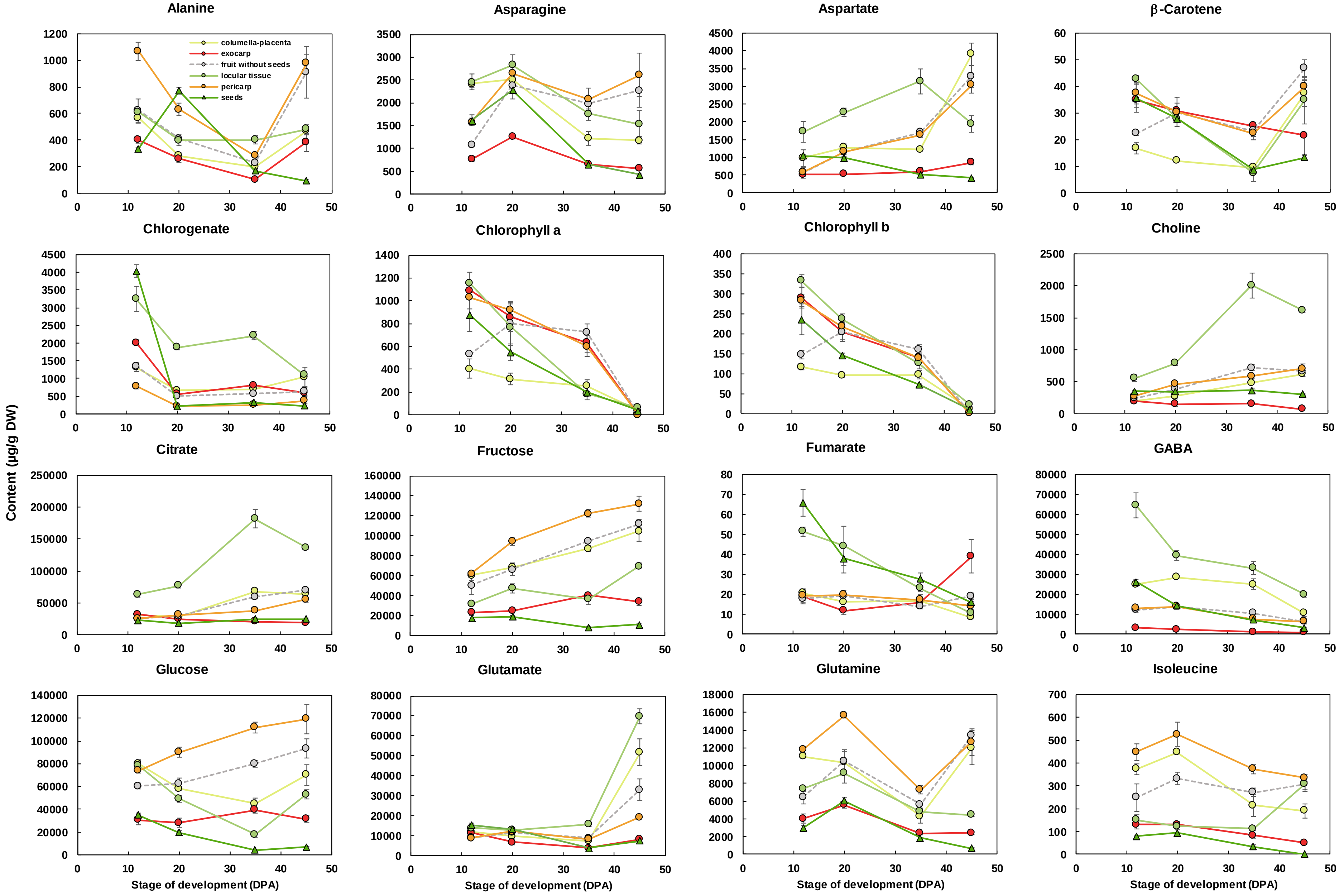

Supplementary Figure S1 continued

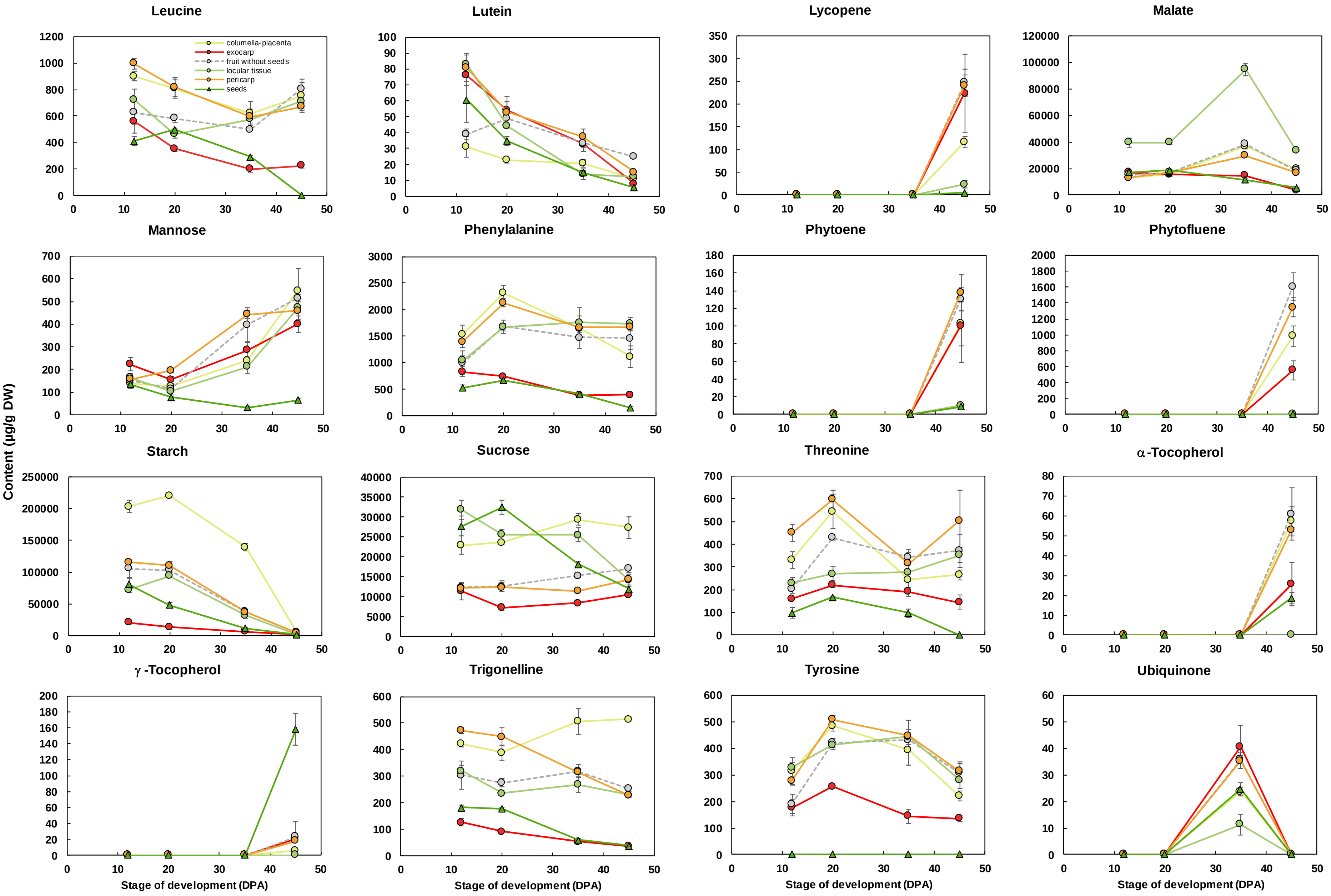

Supplementary Figure S1 continued

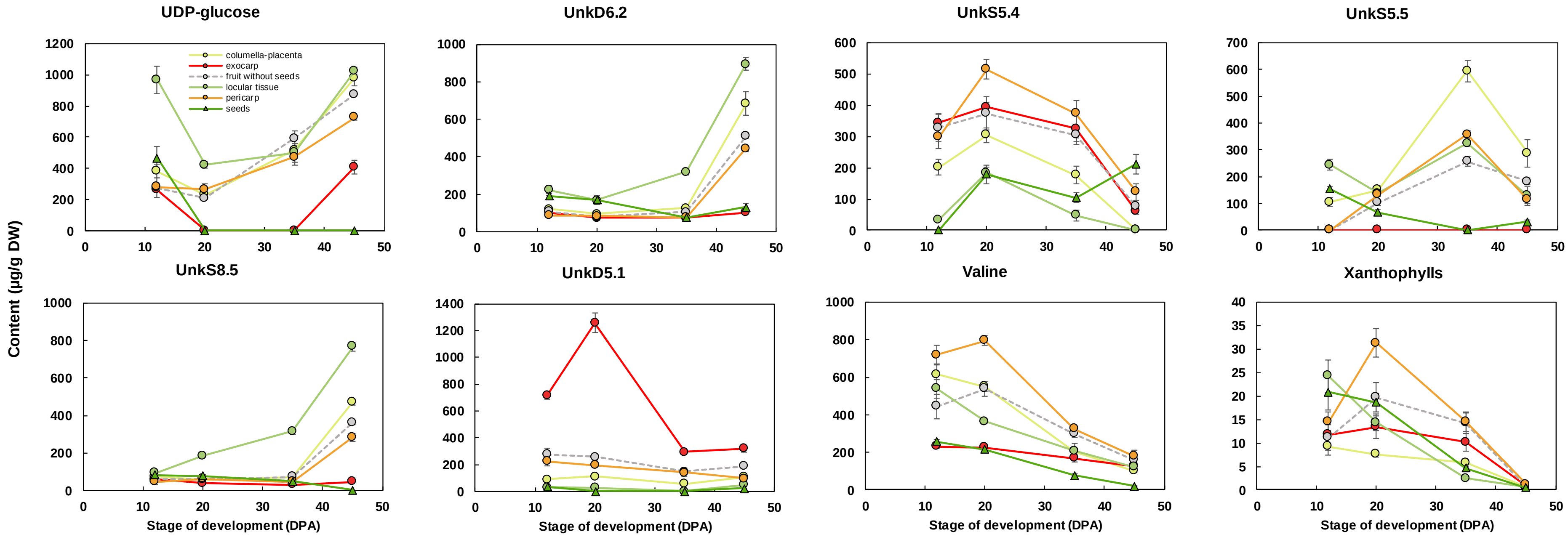

Supplement: Supplementary file 1 [file metabolites-09-00093-s001.zip › Supplementary-Figure S1.pdf]
