# Supplementary material for: NMR-Based Tissular and Developmental Metabolomics of Tomato Fruit
Source: Metabolites. 2019 May 9;9(5):93. doi: 10.3390/metabo9050093 (PMC6571556; doi:10.3390/metabo9050093)
Supplement: Supplementary file 1 [file metabolites-09-00093-s001.zip › Supplementary-Figure S2.pdf]

Supplementary Figure S2: Compositional distances between sample means per tomato fruit tissue and stage of development based on the absolute contents in 39 metabolites and starch. Euclidian distance calculated using mean centred and unit-variance scaled data of five tissues and four stages of development. In bold, distances lower than 0.5 between sample means per tissue and stage mapped in figure 5.

Article Title: NMR-based tissular and developmental metabolomics of tomato fruit. *Authors*: Lemaire-Chamley M., Mounet F., Deborde C., Maucourt M., Jacob D., Moing A.

		Exocarp				Pericarp				Columella				Locular tissue				Seeds			
		12DPA	20 DPA	35 DPA	45 DPA	12 DPA	20 DPA	35 DPA	45 DPA	12 DPA	20 DPA	35 DPA	45 DPA	12 DPA	20 DPA	35 DPA	45 DPA	12 DPA	20 DPA	35 DPA	45 DPA
Exocarp	12 DPA		**0.251**	**0.394**	0.598	**0.498**	0.654	0.585	0.888	0.567	0.652	0.647	0.861	0.617	0.516	0.781	0.808	**0.476**	**0.459**	0.518	0.659
	20 DPA			**0.393**	0.600	0.584	0.666	0.602	0.904	0.611	0.653	0.681	0.910	0.755	0.588	0.829	0.863	0.624	0.513	0.526	0.641
	35 DPA				0.520	0.679	0.758	0.514	0.897	0.637	0.685	0.597	0.880	0.790	0.613	0.787	0.837	0.594	0.500	**0.328**	0.536
	45 DPA					0.809	0.905	0.718	0.680	0.710	0.772	0.707	0.707	0.870	0.709	0.838	0.809	0.662	0.583	**0.483**	0.535
Pericarp	12 DPA						**0.352**	0.555	0.803	**0.382**	**0.490**	0.669	0.868	0.587	0.536	0.853	0.848	0.663	0.555	0.765	0.913
	20 DPA							**0.483**	0.806	**0.425**	**0.420**	0.671	0.900	0.671	0.547	0.863	0.880	0.789	0.664	0.870	1.000
	35 DPA								0.717	**0.478**	**0.489**	**0.419**	0.747	0.686	0.518	0.689	0.735	0.713	0.620	0.670	0.823
	45 DPA									0.747	0.782	0.824	**0.456**	0.914	0.783	0.881	0.732	0.947	0.832	0.943	0.992
Columella	12 DPA										**0.224**	**0.452**	0.758	0.564	**0.402**	0.696	0.748	0.592	**0.475**	0.639	0.798
	20 DPA											**0.449**	0.813	0.676	**0.454**	0.702	0.785	0.713	0.577	0.694	0.842
	35 DPA												0.722	0.663	**0.489**	0.540	0.725	0.651	0.573	0.587	0.756
	45 DPA													0.861	0.761	0.778	0.567	0.866	0.807	0.890	0.946
Locular tissue	12 DPA														**0.369**	0.707	0.826	**0.425**	0.582	0.817	0.975
	20 DPA															0.507	0.691	**0.436**	**0.418**	0.616	0.784
	35 DPA																0.606	0.746	0.708	0.733	0.880
	45 DPA																	0.833	0.784	0.838	0.928
Seeds	12 DPA																		**0.416**	0.563	0.736
	20 DPA																			**0.414**	0.614
	35 DPA																				**0.424**
	45 DPA																				
