# Supplementary material for: NMR-Based Tissular and Developmental Metabolomics of Tomato Fruit
Source: Metabolites. 2019 May 9;9(5):93. doi: 10.3390/metabo9050093 (PMC6571556; doi:10.3390/metabo9050093)
Supplement: Supplementary file 1 [file metabolites-09-00093-s001.zip › SupplementaryText S1.pdf]

Supplementary Text S1: **Tentative annotation of unknown metabolites unkD5.1 and unkD6.2**

Article title: NMR-based tissular and developmental metabolomics of tomato fruit. *Authors*: Lemaire-Chamley M., Mounet F., Deborde C., Maucourt M., Jacob D., Moing A.

1. Methods

Besides the observation of the 1D spectra of the different tissues and stages of Ailsa Craig fruits, 1D and 2D spectra of two independent tomato (*Solanum lycopersicum*) fruit extracts, prepared from 30 mg lyophilized powder of pericarp of cv. Sassari at mature-green and red-ripe stages, were acquired for complementary annotation. The cv. Sassari fruits were grown in a greenhouse and harvested in September 2017.

1D and 2D (COSY, Correlation SpectroscopY; HSQC, Heteronuclear Single Quantum Correlation; HMBC, Heteronuclear Multiple Bond Correlation) spectra of the additional tomato extracts and of pure compounds (rhamnose, AMP) were acquired at 500.162 MHz on a Bruker Avance III spectrometer (Bruker Biospin, Karlsruhe, Germany) using a 5-mm ATMA z-gradient broadband inverse probe flushed with nitrogen gas (BBI). A waiting delay (90 s) for temperature homogenization of the sample in the magnet was used. Automatic locking, tuning, matching, shimming (TopShim) and pulse calibration (pulsecal, determination of the correct 90° pulse length) were performed for each sample.

2D NMR experiments were acquired with non-uniform sampling (NUS). The parameters for COSY NUS experiment were NUS percent at 25% and 256 points, 8 dummy scans, 128 scans, 9.45 µs 90° pulse, spectral width 6,000 Hz, 2K data points in F2 and 256 in F1. The ^{1}H ^{13}C HSQC NUS parameters were NUS percent at 25% and 48 points, 16 dummy scans, 128 scans, 9.49 µs 90° pulse in direct dimension and 14.50 µs 90° pulse in indirect dimension, spectral width 27,671 Hz in indirect dimension and 6,000 in the direct one, 2K data points in the direct dimension and 1K in the indirect one. The ^{1}H ^{13}C HMBC NUS parameters were NUS percent at 25% and 256 points, 16 dummy scans, 128 scans, 9.52 µs 90° pulse in direct dimension and 14.50 µs 90° pulse in indirect dimension, spectral width 30,000 Hz in indirect dimension and 6,000 in the direct one, 2K data points in the direct dimension and 1K in the indirect one. 2D spectra were reconstructed using the recursive multidimensional decomposition (mdd) or the compressed sensing approach (cs) available within the TopSpin software (v3.2, Bruker Biospin, Karlsruhe, Germany).

2. Results

2.1. Tentative annotation of unknown metabolite unkD5.1

NMR spectra of the Ailsa Craig tomato exocarp extracts presented a doublet at 5.1 ppm with a small coupling constant of 0.9 Hz. The most intense doublet was observed on 20 DPA extract spectra. It was still detected on Ailsa Craig pericarp extract spectra. A COSY spectrum of a green tomato pericarp extract (cv. Sassari), prepared with the same protocol, exhibited also this low intensity doublet at 5.1 ppm. Nonetheless, low intensity COSY cross peaks were detected at 4.13 ppm and 1.28 ppm. A HSQC spectrum of this green tomato pericarp extract exhibited also this doublet at 5.1 ppm, correlated with a very low intensity peak at 110.5 ppm. No signal correlated with this proton was detected on a HMBC spectra. Additional HSQC and HMBC spectra of ripe tomato pericarp extract (cv. Sassari), prepared with the same protocol, exhibited also this doublet at 5.1 ppm, but no signal correlated with this proton was detected in the spectra.

Spectra of commercial pure rhamnose (1D ^{1}H spectrum in Figure A and HSQC in Figure B), acquired with the same conditions of solvent and pH gave the information presented in Table I below.

Table I: Chemical shifts of selected protons and carbons of rhamnose.

Metabolite	1D ^1H NMR data	^1H ^{13}C HSQC NMR data
β-rhamnose	H1 D@ 5.11 ppm; J 1.6 Hz<br>H2 DD@ 3.93 ppm; J 3.5 and 1.8 Hz<br>H6 D@ 1.27 ppm; J 6.3 Hz	C1 @ 96.0 ppm
α-rhamnose	H1 D@ 4.87 ppm; J 0.7 Hz<br>H2 DD@ 3.94 ppm; J 4.0 and 0.9 Hz<br>H3 D@ 1.29 ppm; J 5.9 Hz	C1 @ 95.5 ppm

D: doublet; DD: doublet of doublet. J: coupling constant in Hz.

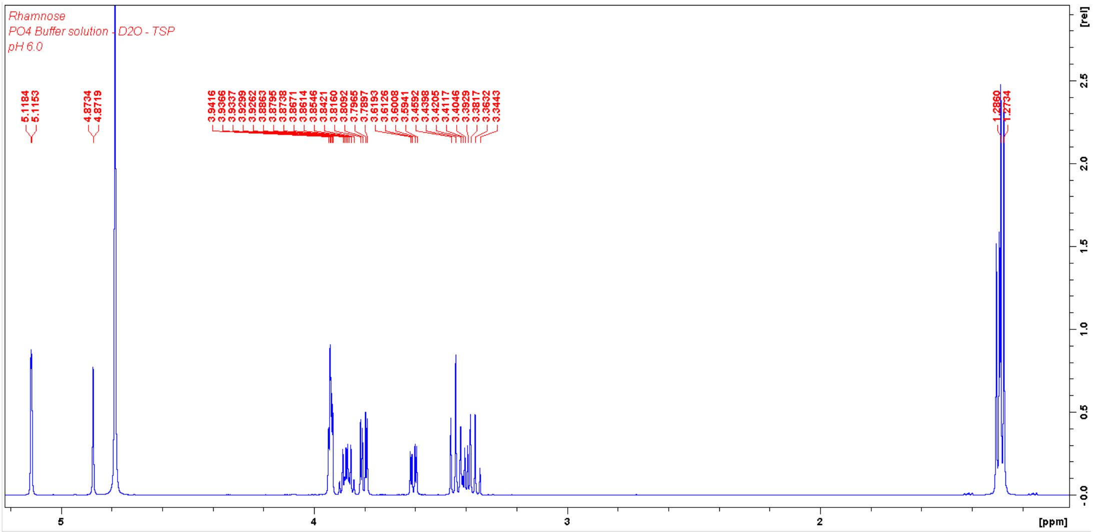

Figure A. 1D ^1H NMR spectrum of rhamnose in 200 mM phosphate buffer solution in D_2O at apparent pH 6.

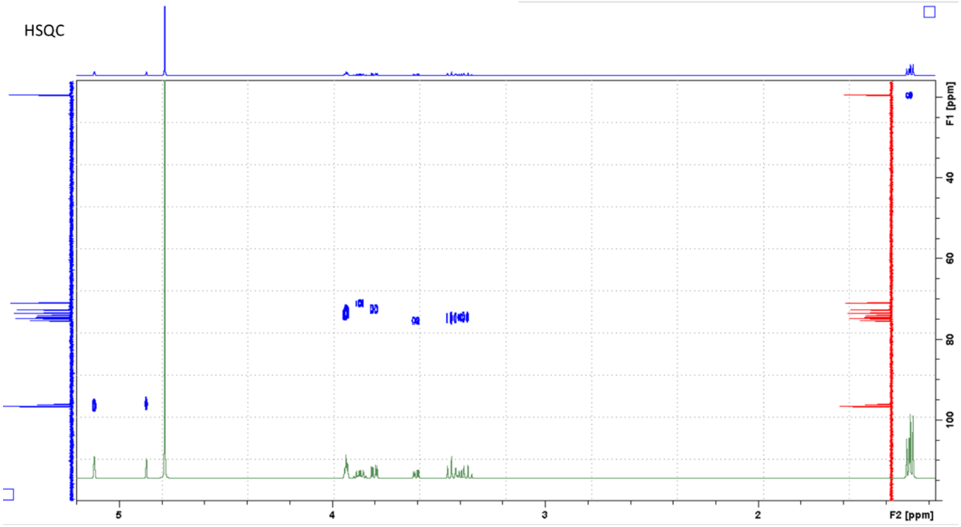

Figure B. 2D ^1H ^{13}C HSQC spectrum of rhamnose in 200 mM phosphate buffer solution in D_2O at apparent pH 6.

If the 5.1 ppm doublet was from free β-rhamnose, its coupling constant would be 1.6 Hz, and a doublet of free α-rhamnose at 4.87 ppm with a coupling constant of 0.7 Hz should be observed which was not the case. However, the signal pattern, a doublet with a small coupling constant 0.9 Hz,

correlated with signals at 4.13 ppm and 1.28 ppm, suggesting that it could be due to a compound with a moiety of α-rhamnosyl (MSI status 3, Sumner et al., 2007). The rutin candidate (α-L-rhamnopyranosyl-(1→6)-β-D-glucopyranose) was excluded, due to the signal at 5.1 ppm with a coupling constant of 7.7 Hz. A preparative extraction with purification steps would be required to identify this compound unambiguously.

2.1 Tentative annotation of unknown metabolite unkD6.2

NMR spectra of the 45 DPA Ailsa Craig tomato pericarp extracts presented a doublet at 6.2 ppm with a coupling constant of 5.9 Hz. A COSY spectrum of a ripe tomato pericarp extract (cv. Sassari), prepared with the same protocol, exhibited also this low intensity doublet at 6.2 ppm. Nonetheless, a low intensity COSY cross peak was detected at 4.77 ppm and a low intensity HSQC correlated peak was detected at 89.5 ppm. No signal correlated with this doublet was detected on the HMBC spectrum. In a previously published work this doublet was assigned to an adenosine-containing compound (Mounet et al., 2007).

Spectra of commercial adenosine-5'-monophosphate (AMP) were acquired in the same conditions of solvent and pH. These spectra are plotted with the tomato extract spectra in Figure C. On the 1D ^{1}H NMR spectrum stack plot (Figure C), the first four patterns of AMP, *i.e.* two singlets at 8.51 and 8.23 ppm, one doublet à 6.13 ppm and one triplet at 4.77 ppm were easily observed on the extract spectrum of 45 DPA Ailsa Craig tomato pericarp and only the first three patterns were observed on the Sassari ripe tomato pericarp spectrum, the last one being under the water signal. On the COSY spectrum (Figure D), the cross peaks of the other peaks of AMP, *i.e.* at 4.77, 4. 55 and 4.38 were detected., The correlated peaks of AMP, *i.e.* at 6.2 - 89; 4.77 - 76.6; 4. 55 - 72.6 and 4.38 - 86.5 were detected on the HSQC spectra of Sassari ripe tomato pericarp extract (Figure E).

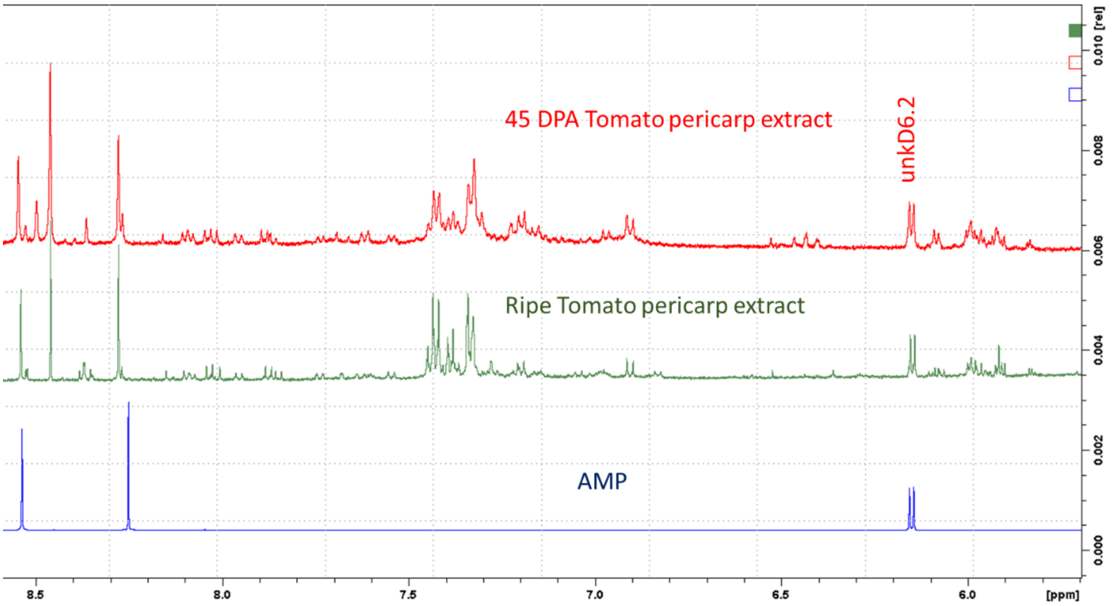

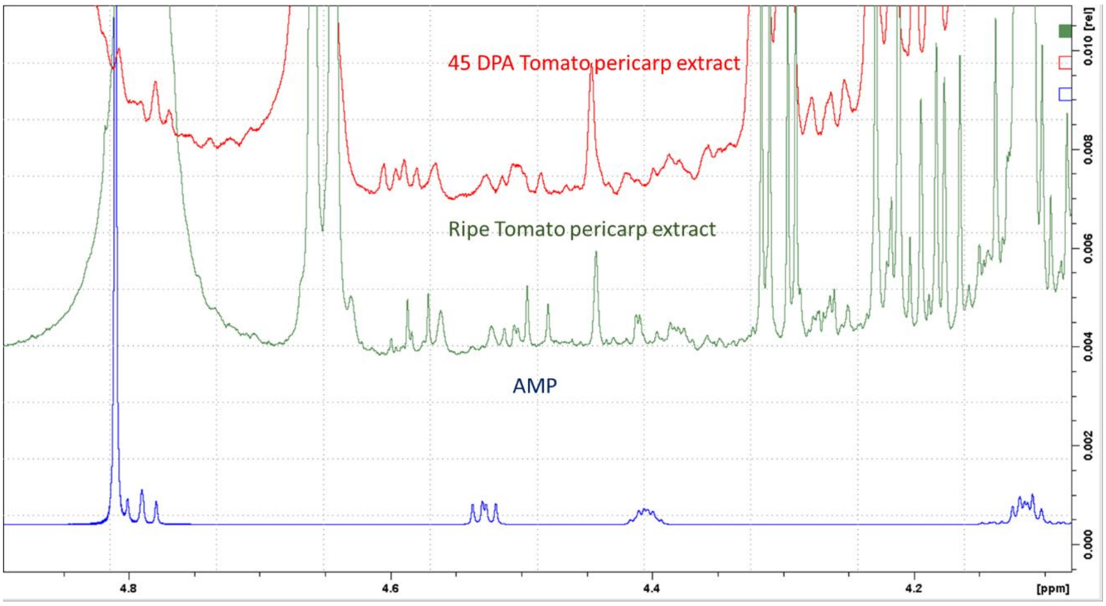

Figure C. 1D ^{1}H NMR spectra of AMP in 200 mM phosphate buffer solution in D_2O at apparent pH 6 (in blue), and of 45 DPA Ailsa Craig (in red) and ripe Sassari (in green) tomato pericarp extracts.

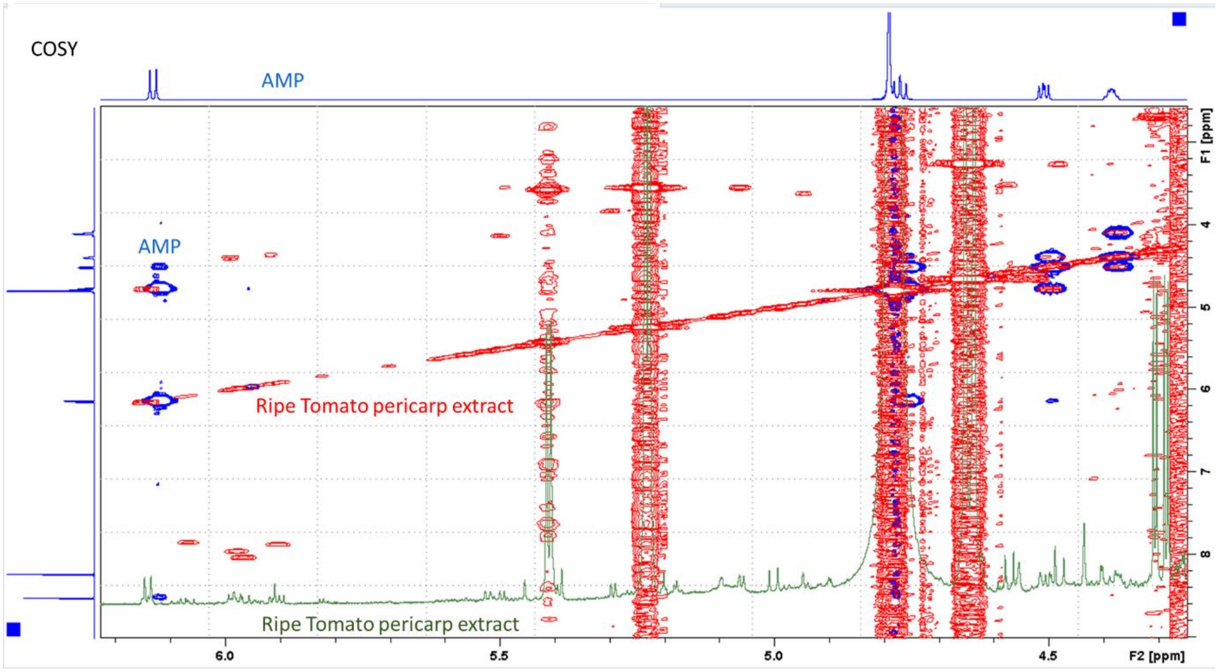

Figure D. 2D ^{1}H-^{1}H COSY NMR spectra of AMP in 200 mM phosphate buffer solution in D_2O at apparent pH 6 (in blue), and of ripe Sassari tomato pericarp extract (1D ^{1}H in green and 2D COSY in red).

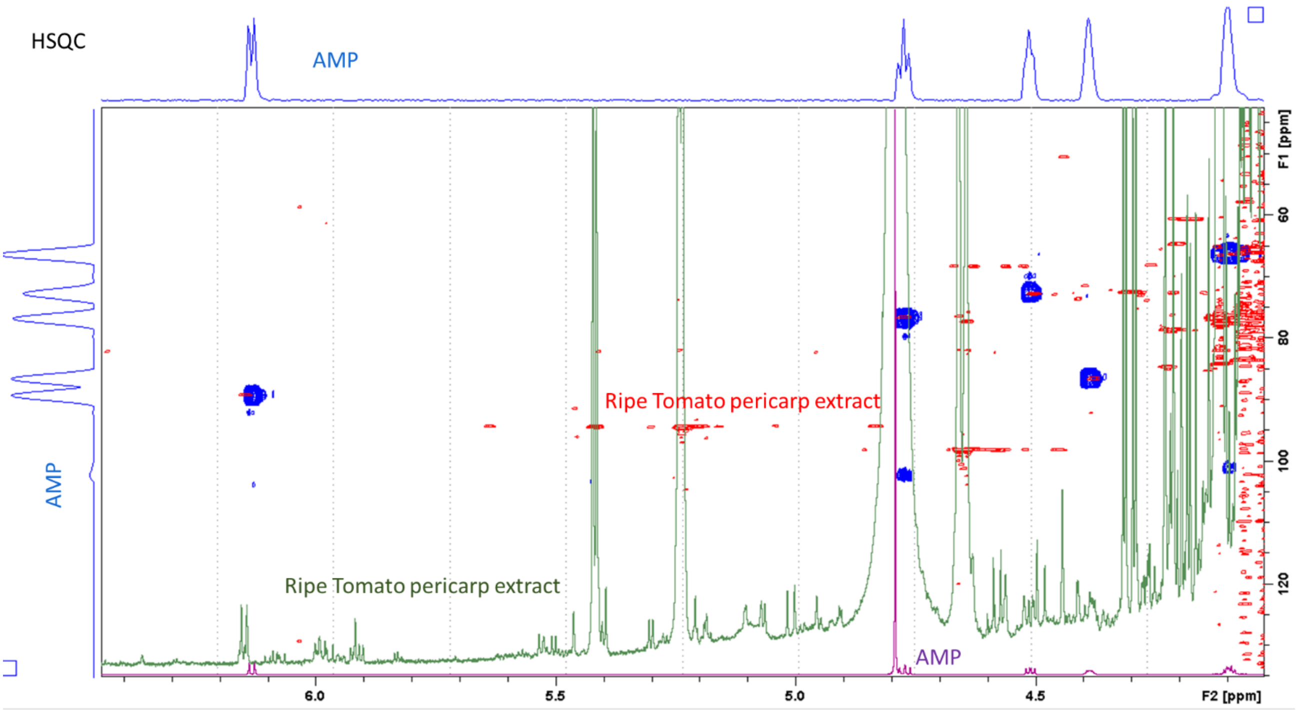

Figure E. 2D ^{1}H-^{13}C HSQC NMR spectra of AMP in 200 mM phosphate buffer solution in D_2O at apparent pH 6 (in blue) and of Sassari ripe tomato pericarp extract (1D ^{1}H in green and 2D HSQC in red).

All these spectral information lead to confirm that the unknown compound with a doublet at 6.2 ppm is adenosine-5'-monophosphate (MSI status 1, Sumner et al., 2007).

References

Mounet, F.; Lemaire-Chamley, M.; Maucourt, M., et al. Quantitative metabolic profiles of tomato flesh and seeds during fruit development: complementary analysis with ANN and PCA. *Metabolomics*. **2007**, *3*, 273-288. doi:10.1007/s11306-007-0059-1.

Sumner, L.W.; Amberg, A.; Barrett, D., et al. Proposed minimum reporting standards for chemical analysis Chemical Analysis Working Group (CAWG) Metabolomics Standards Initiative (MSI). *Metabolomics*. **2007**, 3, 211-221. doi:10.1007/s11306-007-0082-2.
